# Advances and Challenges in Studying Type III Secretion Effectors of Attaching and Effacing Pathogens

**DOI:** 10.3389/fcimb.2020.00337

**Published:** 2020-07-07

**Authors:** Sabrina L. Slater, Gad Frankel

**Affiliations:** MRC Centre for Molecular Bacteriology and Infection, Department of Life Sciences, Imperial College London, London, United Kingdom

**Keywords:** enteropathogenic *E. coli*, EPEC, enterohaemorrhagic *E. coli*, EHEC, T3SS, effector, *C. rodentium*, machine learning

## Introduction

Outbreaks of the diarrhoeal disease caused by enteropathogenic *Escherichia coli* (EPEC) and enterohaemorrhagic *E. coli* (EHEC) present a significant burden to public health in countries with low and high human development indices (HDIs) alike. Over the course of evolution, horizontal gene transfer events have expanded the 4.1 Mb core genome of the human colonic commensal *E. coli* by 1 Mb, resulting in the emergence of pathogens (Croxen and Finlay, 2010; Clements et al., 2012). Each pathogenic strain is characterized by their unique virulence factor repertoire and clinical epidemiology (Nataro and Kaper, 1998; Gomes et al., 2016). EPEC causes infantile diarrhea in countries with a low HDI. Indeed, from 2007 to 2015 the World Health Organization (WHO) estimate 230,000 cases of death from diarrhoeal disease, of which EPEC was responsible for 16%, disproportionately affecting children under five (WHO, 2015). EHEC, on the other hand, is defined by its ability to produce Shiga toxins (Stx) (Melton-Celsa, 2014; Krause et al., 2018). Distinguishing between EPEC and EHEC is clinically important, as treatment of EHEC with antibiotics can incite Stx expression (Zhang et al., 2000) and consequently acute kidney failure, a sequela of haemolytic uremic syndrome (HUS) (Pacheco and Sperandio, 2012).

EPEC and EHEC are united in their ability to intimately adhere to human enterocytes, causing elongation and loss of microvilli, and the formation of actin-rich pedestals at the site of bacterial attachment (Finlay et al., 1992; Frankel and Phillips, 2008) As such, EPEC and EHEC are members of the attaching and effacing (A/E) family of pathogens, which also include *E. albertii* (Bhatt et al., 2019), the murine-restricted *Citrobacter rodentium* (Mullineaux-Sanders et al., 2019) and rabbit enteropathogenic *E. coli* (REPEC) (Milon et al., 1999). The formation of A/E lesions is facilitated by proteins encoded on the locus of enterocyte effacement (LEE), a largely conserved 35.6 kb pathogenicity island that encodes components of the Type 3 Secretion System (T3SS). The T3SS of A/E pathogens is ~3.5 MDa and includes several elements. Its cytoplasmic complex is equipped with an ATPase (EscN), secretion regulators (SepL and SepD) and chaperones (such as CesT). A basal body spans the inner and outer membranes, tethering the sheathed extracellular EscF needle to EspA filaments (Knutton et al., 1998) and culminates in the translocation pore (comprising EspB and EspD) in the host membrane (reviewed by Slater et al., 2018). The integration of signals from the gut environment, the microbiome and chaperones facilitate T3SS assembly, and translocation of effector proteins from the bacterium directly into the host cytosol (McDaniel et al., 1995; Connolly et al., 2015; Furniss and Clements, 2017; Katsowich et al., 2017; Serapio-Palacios and Finlay, 2020).

The effectors of A/E pathogens are encoded on either the LEE, prophages or insertion elements. While all the effectors rely on an N-terminal translocation sequence and specific chaperone-binding motifs to guide secretion (Deng et al., 2017; Slater et al., 2018; Wagner et al., 2018), their sequences are otherwise highly adapted to intercept specific host processes. Additionally, effector functions can be antagonistic or cooperative (Shenoy et al., 2018), which taken together with their low abundance and continuous acquisition, underpins the challenge of identifying and studying effectors in a meaningful context.

Current research into the effector biology of A/E pathogens can be considered in three phases: discovery, *in vitro* functional characterization, and defining the contribution of each effector to the pathogen's infection strategy *in vivo*. To date, 30 families of effectors have been identified in A/E pathogens (Table 1). However, as with many pathogens, the rate of effector discovery has surpassed their biological characterization, and the contribution of many effectors to pathogenesis remains unknown. Here we highlight recent advances in technical and conceptual approaches to characterize effectors in A/E pathogens in the context of human disease.

**Table 1:** Reported repertoire of A/E effectors, their method of discovery and inferred role during infection.

**Effector**	**Discovery method**	**Inferred function during infection**
Cif	Transposon mutagenesis in rabbit ileal loop model	Promotes bacterial survival/ cell cycle arrest
Tir/EspE	Anti-phosphotyrosine blotting	Host adherence, actin polymerisation and pyroptosis
EspF	LEE inspection	Induces apoptosis and disrupts tissue architecture
EspG	LEE inspection/ effector homology	Manipulates small GTPases
EspH	LEE inspection	Cytoskeletal remodeling, inhibition of phagocytosis
EspI/NleA	Coomassie of secreted proteins	Inhibits inflammasome activation and protein secretion
EspJ	Transcriptome analysis of adhered bacteria	Inhibits phagocytosis
EspK	Transposon mutagenesis in calf model	Unknown
EspL	Mass spectrometry (MS) of secreted proteins	Inhibits necroptosis
EspM1/2	MS of secreted proteins	Cytoskeletal remodeling
EspN	MS of secreted proteins	Unknown
EspO	MS of secreted proteins	Promotes tissue integrity by promoting IL-22 secretion
EspR	MS of secreted proteins	Unknown
EspS/Ibe/OspB	Effector homology	Suppresses colonic crypt hyperplasia
EspT	Effector homology	Cytoskeletal remodeling, NF-κB modulation
EspW	MS of secreted proteins	Cytoskeletal remodeling
EspX/NleL	MS of secreted proteins	Ligates ubiquitin
EspY	MS of secreted proteins	Unknown
EspZ/SepZ	Transposon mutagenesis in cell culture	Limits effector translocation
Map	LEE inspection	Cytoskeletal remodeling, mitochondrial disruption and colonic oxygenisation
NleB	MS of secreted proteins	Inhibits pro-inflammatory signaling and necroptosis
NleC	MS of secreted proteins	NF-κB inhibition
NleD	Transposon mutagenesis in bovine gastrointestinal tract model	MAPK signaling inhibition
NleE	MS of secreted proteins	NF-κB inhibition
NleF	MS of secreted proteins	Inhibits caspase-4/8/9 activity to limit cell death
NleG	MS of secreted proteins	Ligates ubiquitin
NleH/OspG	Proximity to known effector	Inhibits cell death and NF-κB signaling
NleJ	MS of secreted proteins	Unknown
NleK	MS of secreted proteins	Unknown
TccP/EspF_U_	Transcriptome analysis of adhered bacteria	Actin polymerisation

## Predicting and Verifying Translocation Substrates

Several resources have been developed to identify new effectors. Effector-encoding genes can be predicted *in silico* to varying degrees of accuracy (McDermott et al., 2011; Hobbs et al., 2016; Xue et al., 2019). These algorithms harness experimental knowledge of typical type III effector features, such as N-terminal enrichment of small polar amino acids (e.g., serine and threonine; Arnold et al., 2009), conservation of regulatory motifs upstream of the gene, a differing GC content to the rest of the genome, lack of gene homology to non-T3SS-encoding strains, and gene proximity to known effectors (Teper et al., 2016). Indeed, many novel type III effectors have been identified and validated using algorithm-based approaches, including *Pseudomonas syringae* and *P. fluorescens* (Vinatzer et al., 2005; Samudrala et al., 2009), *Ralstonia spp*. (Sabbagh et al., 2019), *S*. Typhimurium (Samudrala et al., 2009), *Chlamydia trachomatis* and *C. psittaci* (Hovis et al., 2013), *Xanthomonas euvesicatoria* (Teper et al., 2016) and *Pantoea agglomerans* (Nissan et al., 2018).

Despite these successes, algorithm-based approaches have yet to be applied to A/E genomes. Instead, effectors in A/E pathogens were historically discovered through manually curating pathogenicity island genes and mutagenesis screening (Dziva et al., 2004; Mundy et al., 2004; Kanack et al., 2005), homology searches to other T3SS effectors in different species (Bulgin et al., 2009; Petty et al., 2010), mRNA profiling during infection (Dahan et al., 2005), and peptide discovery mass spectrometry (MS) of secreted proteins, notably in combination with 2D gel electrophoresis and effector hypersecretion mutants (Kresse et al., 2000; Creasey et al., 2003; Deng et al., 2004, 2010, 2012; Gruenheid et al., 2004; O'Connell et al., 2004; Tobe et al., 2006; Orton et al., 2013). Moving forward, the employment of techniques that do not rely on homology offer less bias and are therefore preferable. Additionally, A/E pathogens that encode a second functional T3SS (named ETT2) may also secrete its own cognate effectors (Fox et al., 2020), and secretion substrates could be shared between the two T3SSs, as there is evidence for regulatory crossover (Zhang et al., 2004; Luzader et al., 2016).

Once identified, the T3SS-dependent translocation of candidate effectors must be experimentally confirmed. A common approach, developed in 2004 by Charpentier and Oswald, is to C-terminally tag the effector with the TEM-1 β-lactamase and infect CCF2-loaded cells (Charpentier and Oswald, 2004); alternative and refined protocols have since been developed that decrease the tag size, minimize cell toxicity and offer single cell resolution. Collectively, these approaches benefit from their capacity to support different modes of analysis depending on the infection setup, such as enzymatic assays, optical readouts in a 96-well plate, flow cytometry and immunofluorescence microscopy (Mills et al., 2008; Miyake et al., 2008; Gawthorne et al., 2016; O'Boyle et al., 2018).

## *In vitro* Characterization of the Effectors

Approaches to characterize the role of each new effector during infection are ever-developing. At its most fundamental, effector functionality can be investigated under overexpression conditions *in vitro*, where amenable cells are transfected for ectopic effector expression or microinjected with purified protein. Overexpression protocols can provide readouts for drastic visual phenotypes, such as the radical cytoskeletal rearrangements resulting from the transfection of EspV (Arbeloa et al., 2011). Non-mammalian eukaryotic systems such as *Saccharomyces cerevisiae* have also been instrumental in the definition of eukaryotic interaction partners for A/E effectors (Hardwidge et al., 2004; Popa et al., 2016), as well as delineating interfaces for substrate interaction and catalytic residues (Blasche et al., 2013, 2014; Sandu et al., 2017).

These approaches, however, share a common weakness: effectors localize differently when not natively translocated through the injectisome. As such, the infection of appropriate mammalian cells (i.e., colonic epithelial cells) with bacteria translocating a tagged effector protein can provide a more physiologically relevant readout for effector localization. Indeed, native expression of a tagged effector is readily achievable by introducing a C-terminal tag onto the chromosome for effector visualization by immunofluorescence, or for use in tandem with co-immunoprecipitation and/or MS to probe for host protein interactors upon infection. Chromosomal manipulation by triparental conjugation works efficiently in A/E pathogens and other enteric pathogens (Mullineaux-Sanders et al., 2017; Watson et al., 2019; Wong et al., 2019). This conjugation protocol can similarly be used to generate scarless isogenic effector mutants in lieu of traditional gene disruption with antibiotic resistance cassettes or transposon elements (Cepeda-Molero et al., 2017).

## Defining Roles for the Effectors During Infection

Despite the ease of culturing, transfection and microscopy offered by non-polarized and non-colonic cells, the integrated use of more relevant models circumvents cell-line-specific phenotypes. A shift in practice toward more native models for infection is therefore evolving, using differentiated, polarized colonic cell lines, explants and organoids, primary tissues and laboratory animals (Carvalho et al., 2005; Law et al., 2013; Lewis et al., 2016; Cepeda-Molero et al., 2017). Increasingly, infections with A/E pathogens are also modeled in immune-associated cells, whose distinctive protein expression profiles allows researchers to probe the impact of effector delivery on immune-specific pathways (Pearson et al., 2017; Goddard et al., 2019), providing an alternative insight into the impact of effectors in human infection.

One particularly useful model for probing effector function is the infection of mice with *C. rodentium. C. rodentium* is a natural murine pathogen which causes transmissible colonic crypt hyperplasia (CCH) and A/E lesions that are indistinguishable from those cause by EPEC and EHEC in humans (Barthold et al., 1978). Critically, *C. rodentium* shares 67% homology with EPEC and EHEC genomes, most notably in the LEE (Petty et al., 2010, 2011), making it an invaluable tool for the study of the role of the T3SS and its cognate effectors *in vivo* (Mundy et al., 2005; Borenshtein et al., 2008; Collins et al., 2014; Mullineaux-Sanders et al., 2019).

The contribution of single or multiple effectors to pathogenesis can be assayed *via* infection with *C. rodentium* deletion or point mutants (Crepin et al., 2016). Key to revealing novel and physiological phenotypes is the selection of an appropriate mouse strain, or knock-out mice (Simmons et al., 2002; Zheng et al., 2008; Carson et al., 2019). To highlight some examples, the mouse model has delineated Tir, NleA and NleB as essential effectors for efficient colonization (Deng et al., 2003; Mundy et al., 2004; Kelly et al., 2006), demonstrated the impact of individual effectors deletions on host physiology, such as EspO and EspS impacting CCH (Berger et al., 2018; Connolly et al., 2018), and substantiated *in vitro* data indicating Map impacts colonic oxygen availability through mitochondrial disruption (Berger et al., 2017). Recently, mouse-specific differences in infection signatures have been identified through RNAseq and proteomics (Kang et al., 2018; Carson et al., 2019); it remains to be seen whether these differences are fine-tuned by the synergistic action of the effectors.

There are important genetic differences between *C. rodentium* and human A/E pathogens. While *C. rodentium* encodes a type IV pilus named colonization factor *Citrobacter* (CFC), which is related to the EPEC bundle forming pilus (BFP) (Mundy et al., 2003), it does not encode some strain-specific effectors, nor does it express the Shiga toxin or a flagellum, and it likely benefits from mouse-specific host adaptations. Nevertheless, modifications to this model can be implemented to investigate specific aspects of infection by human A/E pathogens, such as HUS and diarrhea (Vallance et al., 2003; Mallick et al., 2012), and drawing parallels from *C. rodentium* studies offers invaluable insight into human infections.

At the other end of the spectrum, the use of minimal effector models should prove instrumental for delineating the complex interplay between effectors, where the creation and complementation of isogenic strains cannot. As has been employed for *Yersinia pestis* (Palace et al., 2018), all known effectors were recently removed from the EPEC E2348/69 genome to investigate the contribution of select effectors and intact pathogenicity islands to A/E lesion formation on the human intestinal mucosa *ex vivo*, confirming that while Tir is essential, it is not sufficient and other elusive effectors are required (Cepeda-Molero et al., 2017). Undoubtedly the same approach would be of great use for other T3SS-encoding pathogens. Building on the decades of fundamental biochemical research into individual effector proteins, the mutation of clusters of effectors with similar functions *in vitro* should also be considered.

Despite the wealth of technical and biological knowledge unearthed over the last two decades, the synergies and redundancies in the function of the effectors hamper the comparison of *in vitro* research to *in vivo* scenarios. However, large-scale sequencing efforts of human pathogenic *E. coli* isolates have revealed the correlation between the presence of effectors, and other virulence factors, and the severity of human pathology through comparative genomics (Donnenberg et al., 2015; Hazen et al., 2016). This can be extended to assaying the prevalence of effectors in strains from different environments (Xu et al., 2017). Although challenging to integrate, these datasets offer unparalleled insight into the relative importance of effector proteins during human infection. Finally, following translocation, effectors form tight interaction networks. As clinical EPEC and EHEC isolates encode strain-specific effector gene combinations, it would be interesting to test the robustness of these networks *in vivo*. This will offer insight into the virulence potential of alternative effector combinations present in clinical isolates. *C. rodentium* provides an ideal model to address this.

## Conclusions and Future Perspectives

With technological advances, our conceptual approaches must keep up. For example, moving beyond the idea that effectors act exclusively once translocated has illuminated greater functionality for *C. rodentium* NleB, which was recently demonstrated to GlcNAcylate an intrabacterial glutathione synthase GshB to promote bacterial survival (El Qaidi et al., 2020); other effectors with enzymatic functions may also have intrabacterial targets. In the same vein, addressing effector functionality in a top-down fashion (from mammalian phenotype to bacterial genotype) has recently unveiled both a unique mode of EPEC-induced inflammatory cell death and a further role for Tir during infection (Goddard et al., 2019).

Effector proteins exhibit fascinating diversity and specificity. Consequently, their study is warranted not only by their importance in human disease. Effectors can demonstrate first-in-class functions, the characterization of which broadens our understanding of protein biochemistry. Additionally, as effectors have witnessed the complexity of eukaryotic signaling cascades for considerably longer than modern researchers, they offer a unique insight into the breadth of function our own cells.

## Author Contributions

SS and GF wrote the manuscript. All authors listed have made a substantial, direct and intellectual contribution to the work, and approved it for publication.

## Conflict of Interest

The authors declare that the research was conducted in the absence of any commercial or financial relationships that could be construed as a potential conflict of interest. The handling editor declared a past collaboration with the author GF.
